# Combinations of soil properties, carbon inputs and climate control the saturation deficit dynamics of stable soil carbon over 17-year fertilizaiton

**DOI:** 10.1038/s41598-018-31028-x

**Published:** 2018-08-23

**Authors:** Jiaying Di, Minggang Xu, Wenju Zhang, Xiaogang Tong, Xinhua He, Hongjun Gao, Hua Liu, Boren Wang

**Affiliations:** 1 National Engineering Laboratory for Improving Quality of Arable Land, Institute of Agricultural Resources and Regional Planning, Chinese Academy of Agricultural Sciences, Beijing, 100081 China; 2 Key Laboratory of Agro-information Services Technology of Ministry of Agriculture, Agricultural Information Institute, Chinese Academy of Agricultural Sciences, Beijing, 100081 China; 3 College of Resources and Environment, Northwest Sci-Tech University of Agriculture and Forestry, Yangling, Shannxi 712100 China; 4 College of Resources and Environment, Southwest University, Chongqing, 400715 China; 5 School of Biological Sciences, University of Western Australia, Crawley, WA 6009 Australia; 6 Institute of Agricultural Resource and Environment, Jilin Academy of Agricultural Sciences, Changchun, 130033 China; 7 Institute of Soil, Fertilizer and Agricultural Water Reduction, Xinjiang Academy of Agricultural Sciences, Urumqi, 830091 China

## Abstract

The soil organic carbon (SOC) saturation deficit (C_sd_) of silt and clay fractions represents the potential for SOC sequestration in a stable form and can influence organic C stabilization efficiency. Little is known, however, about temporal changes of stable soil C_sd_ and how it is affected by soil properties, climate and C inputs. We investigated the temporal changes in the C_sd_ of fine fractions (<53 μm) and examined the factors controlling these changes at three dry-land sites with 17-year fertilizer management histories in China. The rates of change in the stable soil C_sd_ under manure treatments varied from −0.72 to −1.24% yr^−1^ after 17 years of fertilization, indicating that stable C levels under manure treatments were significantly higher than those under other treatments. Stable soil C_sd_ was controlled by a combination of soil properties, temperature, and C inputs at all sites, and the higher variance of C_sd_ of fine fractions can be explained by the soil properties (up to 50%). Furthermore, the quantity of C inputs was the most influential variable for stable soil C_sd_. These results revealed key controls on stable C sequestration potential and indicated the need to develop management strategies to promote stable C sequestration under long-term intensive fertilization.

## Introduction

Maintaining or enhancing soil organic carbon (SOC) is important for improving soil quality and mitigating carbon dioxide (CO_2_) emissions^1–3^. In agricultural ecosystems, cropland management practices such as fertilizer application, crop rotation, plant residue return, and manure inputs can be used to increase SOC stocks and thus enhance soil quality. Various field experiments investigating the effects of agricultural practices on soil carbon changes have been performed^4–9^. However, previous studies found that no extra C was sequestered in the bulk soil of highly aggregated soils^10^, particularly in the stable fractions under long-term high C inputs^11–14^, which indicates SOC saturation. Soil C saturation suggests that with increasing C inputs, the SOC stock will reach a maximum, and the SOC accumulation rate will decrease during this process^15–17^. Hence, SOC saturation should be considered; otherwise, SOC model simulations or SOC potential prediction might generate considerable uncertainty.

The capacity of soils to stabilize SOC is determined by the silt and clay contents of the soil^18–20^. When the upper limit for the adsorption of organic C inputs to clay and silt fractions was reached, increasing the C inputs did not lead to any further increase in C associated with fine fractions^18^. The difference between the saturated organic C content in the fine fractions and the actual measured organic C content of these fractions is referred to as the stable soil C saturation deficit (i.e., the potential for soil silt and clay particles  to sequester additional organic C)^15,18,21^. This soil C saturation deficit affects the ability of soils to store C inputs in a stable form. Several experimental studies have estimated C saturation deficit and found that there were significant differences among soils under different land uses (e.g., croplands, forest, and grassland) on regional scales^21–27^. The stable soil C saturation deficit was found to be greatly affected by climate (temperature and precipitation), soil physicochemical properties, or terrain at the regional scale^21,26,27^. However, the potential for soil C sequestration is jointly controlled by environmental variables, including land management, soil physical and chemical properties, and climate^16^. Therefore, a study focusing on the effect of a single factor to predict stable C saturation deficit changes would be insufficient.

In agricultural systems, the quantity and quality of C inputs associated with crop types or agricultural practices such as fertilization, manure amendments, and straw return are believed to affect SOC stabilization and consequently influence stable soil C saturation deficit due to their different decomposition rates^7,28,29^. Moreover, the temporal changes in SOC were significantly affected by fertilization management^5,30,31^. The relative importance of climate, soil properties, C inputs and C pools and their complex interconnections in regulating temporal SOC dynamics were investigated under different agroecosystems across the Australian cropping regions^32^. There is a need to quantify the effects and contributions of the dominant regulating factors (i.e., climate, inherent soil properties, and quality and quantity of C inputs) on the stable soil C saturation deficit in cropland soils. It is not well understood how the stable soil C saturation deficit temporally changes with fertilization time under different fertilization treatments. The dominant controls on stable soil C saturation deficit dynamics in typical croplands are also unknown.

The objectives of this study were to examine the temporal changes in the stable soil C saturation deficit and to determine the relative contributions of soil properties, C inputs, and climate to the stable soil C saturation deficit. We selected soils from three long-term agricultural soil fertility sites from 1990 to 2007 in northeast, northwest, and southern China. There is a variety of fertilizer treatments (e.g., chemical fertilizer, straw return, and manure amendment) at these three sites, resulting in different quantities and qualities of C inputs to soils. These experimental trials allowed us to test how these fertilizer treatments impact the temporal changes in the stable soil C saturation deficit.

## Results

### Mass proportion and C saturation of silt and clay fractions under different treatments

Silt and clay fractions comprised a large mass proportion of the bulk soil (0.59–0.90). The average mass proportion of the silt and clay fractions was significantly lower at Urumqi (0.73) than at Gongzhuling and Qiyang (0.89 and 0.88, respectively) (Table 1). The average stable soil C saturation was 28.4 g kg^−1^at Qiyang, which was significantly lower than those at Gongzhuling (33.5 g kg^−1^) and Urumqi (30.1 g kg^−1^).

**Table 1:** The average mass proportion and soil C saturation value (C_sat_) of silt and clay fractions for topsoils (0–20 cm) under the seven treatments at the three long-term experimental sites.

	Treatments	Gongzhuling	Qiyang	Urumqi
Mass proportion	CT	0.89a (0.01)	0.89a (0.00)	0.70d (0.01)
	N	0.90a (0.01)	0.88a (0.04)	0.74c (0.03)
	NP	0.89a (0.02)	0.87a (0.01)	0.74c (0.00)
	NPK	0.88a (0.02)	0.89a (0.01)	0.81a (0.01)
	NPKS	0.90a (0.01)	0.88a (0.01)	0.59e (0.02)
	NPKM	0.89a (0.00)	0.86a (0.01)	0.76bc (0.00)
	hNPKM	0.88a (0.04)	0.87a (0.01)	0.78ab (0.01)
Average		0.89a (0.01)	0.88a (0.02)	0.73b (0.07)
Average C saturation value (g kg^−1^)		33.5a (0.30)	28.4c (0.52)	30.1b (1.46)

Different lowercase letters indicate that values are significantly different among treatments within a site or that average values are significantly different among study sites at *P* < 0.05. Data in parentheses are the standard error (*n* = 3 at the Qiyang site and *n* = 4 at the Gongzhuling and Urumqi sites for each treatment). CT, non-fertilized control; N, mineral nitrogen; NP, mineral N and phosphorus; NPK, mineral N, phosphorus, and potassium combination; NPKS, NPK plus crop straw return; NPKM, NPK plus livestock manure; hNPKM, higher rates of mineral fertilizer and manure input.

### C inputs under different treatments

**Figure 1:**
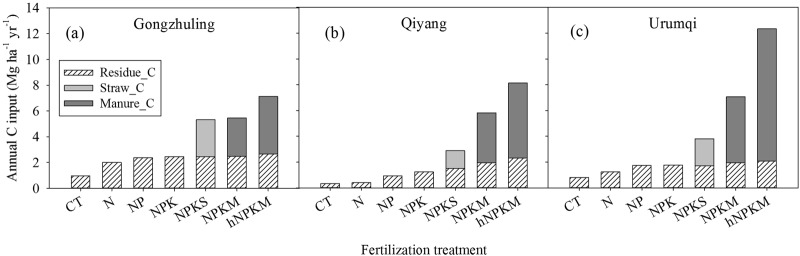
Average annual C input from crop residues, straw return, and manure amendments in various fertilizer treatments at the three long-term sites. CT, non-fertilized control; N, mineral nitrogen; NP, mineral N and phosphorus; NPK, mineral N, phosphorus, and potassium combination; NPKS, NPK plus crop straw return; NPKM, NPK plus livestock manure; hNPKM, higher rates of mineral fertilizer and manure input.

The average annual C input from crop residues under CT was 0.96 Mg ha^−1^ yr^−1^, 0.33 Mg ha^−1^ yr^−1^, and 0.81 Mg ha^−1^ yr^−1^ at Gongzhuling, Qiyang, and Urumqi, respectively (Fig. 1). Applications of mineral fertilizers significantly increased the crop biomass; hence, the annual C input from residues at the three sites increased (0.40–2.46 Mg ha^−1^ yr^−1^). Obviously, NPKS, NPKM, and hNPKM treatments had much higher annual C input than the mineral fertilizer treatments owing to the additional straw or manure. The annual C input under the NPKS treatment was 1.9–2.2 times that under the NPK treatment at all three sites. The highest annual C input at each site was 7.14 Mg ha^−1^ yr^−1^, 8.13 Mg ha^−1^ yr^−1^, and 12.3 Mg ha^−1^ yr^−1^ under hNPKM at Gongzhuling, Qiyang, and Urumqi, respectively (Fig. 1).

### Changes in stable soil C saturation deficit

**Figure 2:**
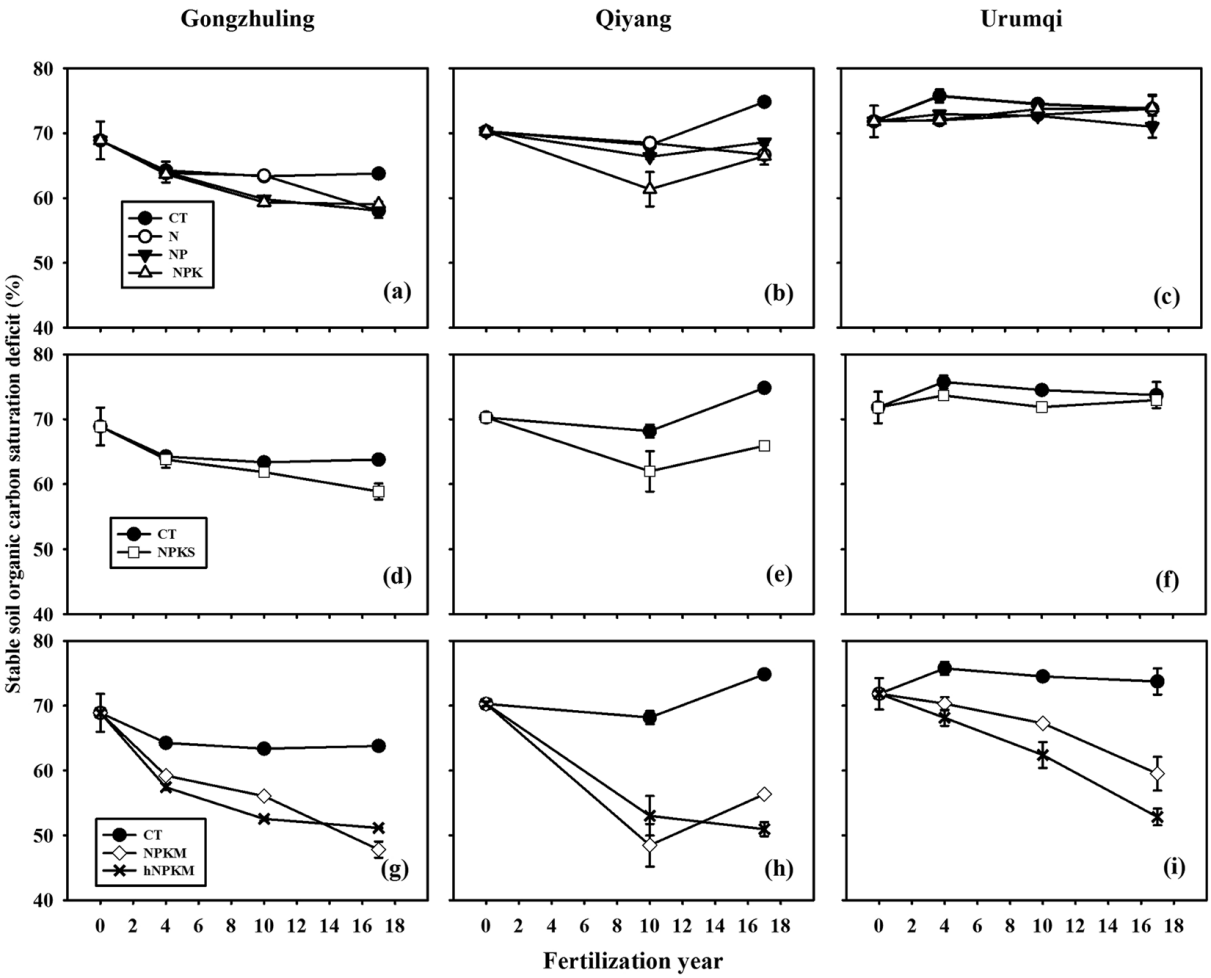
Temporal changes in the stable soil C saturation deficit in the top 20-cm soil layer the following various fertilizer treatments: CT, N, NP, and NPK treatments (**a**–**c**), CT and NPKS (**d**–**f**) and CK, NPKM, and hNPKM (**g**–**i**) at the three long-term experimental sites. All data are expressed as the mean plus standard deviation. CT, non-fertilized control; N, mineral nitrogen; NP, mineral N and phosphorus; NPK, mineral N, phosphorus, and potassium combination; NPKS, NPK plus crop straw return; NPKM, NPK plus livestock manure; hNPKM, higher rates of mineral fertilizer and manure input.

In 1990, the initial stable soil C_sd_ values at Gongzhuling, Qiyang, and Urumqi were 68.9%, 70.3%, and 71.8%, respectively. In 2007, after 17 years of manure amendment, the stable soil C_sd_ values were 47.8%, 56.3%, and 59.5% under NPKM and 51.2%, 51.0%, and 53.3% under hNPKM at Gongzhuling, Qiyang, and Urumqi, respectively (Fig. 2), and these values were smaller than those of the other treatments (*P* < 0.05).

At Gongzhuling, after 4 years of fertilization, the stable soil C_sd_ did not differ significantly among CT, N, NP, NPK, and NPKS treatments (*P* > 0.05, Fig. 2a,d), and the stable soil C_sd_ of all five treatments decreased to 64.0%, with an average annual rate of change (hereafter change rate) of −1.23% yr^−1^(Table 2). In the fourth to the tenth year of the experiment, the average change rates of stable soil C_sd_ values under the N, NP, NPK, and NPKS treatments were −0.06, −0.70, −0.72 and −0.32% yr^−1^, respectively (Table 2). These rates were lower than those in the first four years of fertilization. After 17 years of fertilizer treatment, the stable soil C_sd_ values under N, NP, NPK, and NPKS were not significantly different from each other, with an average of 58.4% (Fig. 2a,d). Stable soil C_sd_ values under NPKM and hNPKM decreased with fertilization time, at 47.8 and 51.1% (Fig. 2g), respectively, and the average change rates of C_sd_ under NPKM (−1.24% yr^−1^) and hNPKM (−1.04% yr^−1^) after 17 years of fertilization were higher than those under the other treatments (Table 2).

**Table 2:** Annual change rates of stable soil C_sd_ among the treatments at the three sites.

Site	Fertilization years (yr)	Annual change rate of C saturation deficit (% yr^−1^)						
		CT	N	NP	NPK	NPKS	NPKM	hNPKM
Gongzhuling	0~4	−1.16	−1.26	−1.22	−1.30	−1.27	−2.42	−2.87
	4~10	−0.15	−0.06	−0.70	−0.72	−0.32	−0.53	−0.81
	10~17	0.06	−0.78	−0.25	−0.04	−0.43	−1.18	−0.20
	0~17	−0.30	−0.64	−0.64	−0.58	−0.59	−1.24	−1.04
Qiyang	0~10	−0.21	−0.18	−0.39	−0.89	−0.83	−2.18	−1.73
	10~17	0.95	−0.26	0.32	0.73	0.56	1.12	−0.29
	0~17	0.27	−0.21	−0.10	−0.22	−0.26	−0.82	−1.14
Urumqi	0~4	0.98	0.04	0.28	0.05	0.46	−0.38	−0.93
	4~10	−0.21	0.14	−0.04	0.28	−0.30	−0.50	−0.96
	10~17	−0.11	0.13	−0.24	0.03	0.16	−1.11	−1.36
	0~17	0.11	0.11	−0.05	0.12	0.07	−0.72	−1.12

CT, non-fertilized control; N, mineral nitrogen; NP, mineral N and phosphorus; NPK, mineral N, phosphorus, and potassium combination; NPKS, NPK plus crop straw return; NPKM, NPK plus livestock manure; hNPKM, higher rates of mineral fertilizer and manure input.

At Qiyang, after 10 years of fertilization, the stable soil C_sd_ under the CT, N, and NP treatments showed a slight decrease compared to the initial values (Fig. 2b, Table 2), and the stable soil C_sd_ values under the NPK, NPKS, NPKM, and hNPKM treatments were 61.4, 62.0, 48.5, and 53.0%, respectively; all of these values were significantly decreased compared to the initial values (*P* < 0.05, Fig. 2b,e and h), with average change rates of −0.89, −0.83, −2.18, and −1.73% yr^−1^, respectively (Table 2). However, in 2007, after 7 years of fertilization, the stable soil C_sd_ under CT, NP, NPK, and NPKM treatments increased compared to the corresponding values in 2000. These increases were related to decreases in the organic C content of mineral soil fractions in these treatments (Fig. 2b,e and h; Table 2).

At Urumqi, after 4 years of fertilization, the stable soil C_sd_ under CT increased slightly, but no real change occurred until after 17 years of fertilization (Fig. 2c). During the 17 years of fertilization, the stable soil C_sd_ did not change over time under the treatments with mineral fertilizers and NPKS (Fig. 2c, f), but it decreased significantly under the NPKM and hNPKM treatments (*P* < 0.05, Fig. 2i). The average change rates of stable soil C_sd_ under the NPKM and hNPKM treatments (−0.72 and −1.12% yr^−1^, respectively) were higher than those under the other treatments after 17 years of fertilization (Table 2).

### Stable soil C saturation deficit  was affected by soil properties, C inputs, and climate

**Figure 3:**
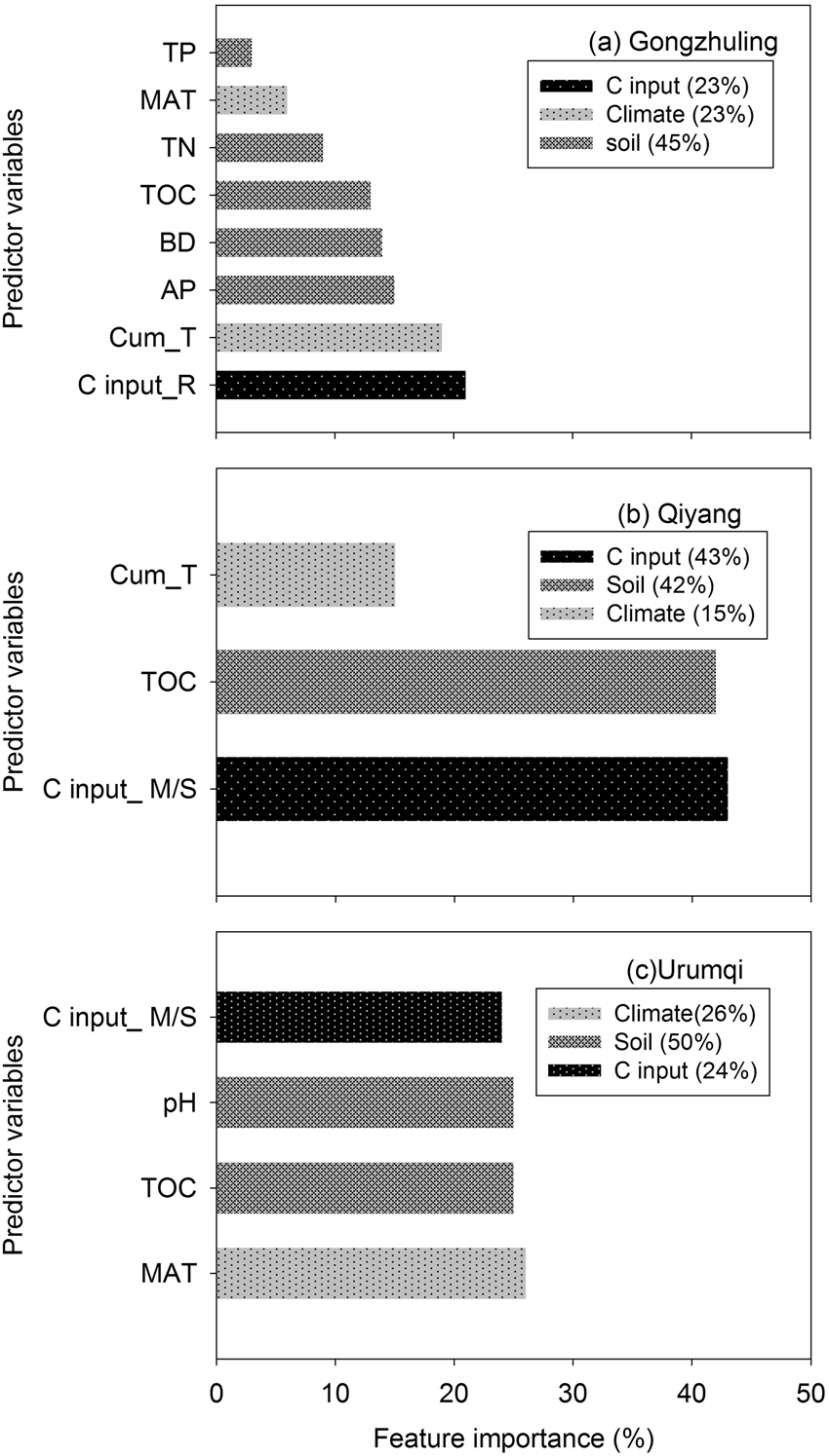
The relative contribution (%) of the variables obtained according to the feature importance of the lasso regression model of stable soil C saturation deficit for Gongzhuling, Qiyang and Urumqi. TOC, total soil organic content; TN, total soil nitrogen; TP, total phosphorus; AP, available phosphorus; BD, bulk density; pH, soil pH; MAT, mean annual temperature; Cum_T >10 °C, effective cumulative temperature >10 °C; C input_R, crop residue-C input amount; C input_M/S, the extra organic C input amount (i.e., manure or straw).

A lasso regression analysis showed that the combination of soil properties, C inputs and climate contributed to stable soil C_sd_ values (Fig. 3). At Gongzhuling, the crop residue-C input level, cum_T >10 °C and available phosphorus (AP) were the three most influential variables on stable soil C_sd_ and together accounted for 69% of the overall influence of all assessed variables (Fig. 3a). Moreover, the relative individual influence of soil properties was small. However, the overall contribution of soil properties to stable soil C_sd_ was 45%, while the values were 23% and 23% from C inputs and climate, respectively (Fig. 3a). At Qiyang, the extra organic C input amount, total organic carbon (TOC) content and cum_T >10 °C were the variables influencing stable soil C_sd_. The overall contribution to stable soil C_sd_ was 43%, 42% and 15% from C inputs, soil properties and climate, respectively (Fig. 3b). At Urumqi, the mean annual temperature (MAT), TOC, pH and extra organic C input amount were the variables influencing stable soil C_sd_, and the contribution to stable soil C_sd_ was 26%, 25%, 25% and 24%, respectively (Fig. 3c). Overall, the lasso regression model driven by the assessed variables shown in Fig. 3 could explain 89%, 77% and 94% of the variation in stable soil C_sd_ for soils in Gongzhuling, Qiyang and Urumqi (Fig. S1), respectively.

## Discussion

The change rates of the stable soil C_sd_ at the three study sites varied among the fertilizer treatments over 17 years (Table 2), demonstrating that fine soil fractions under manure treatments had higher C sequestration rates than those under CT, mineral fertilizer, and straw return treatments. Additionally, the fine soil fractions of manure treatments sequestered more C. The results also suggest that soils under CT, mineral fertilizer, or straw return treatments still have the potential to sequester a large amount of C input due to their high stable soil C_sd_ after 17 years of fertilization.

**Figure 4:**
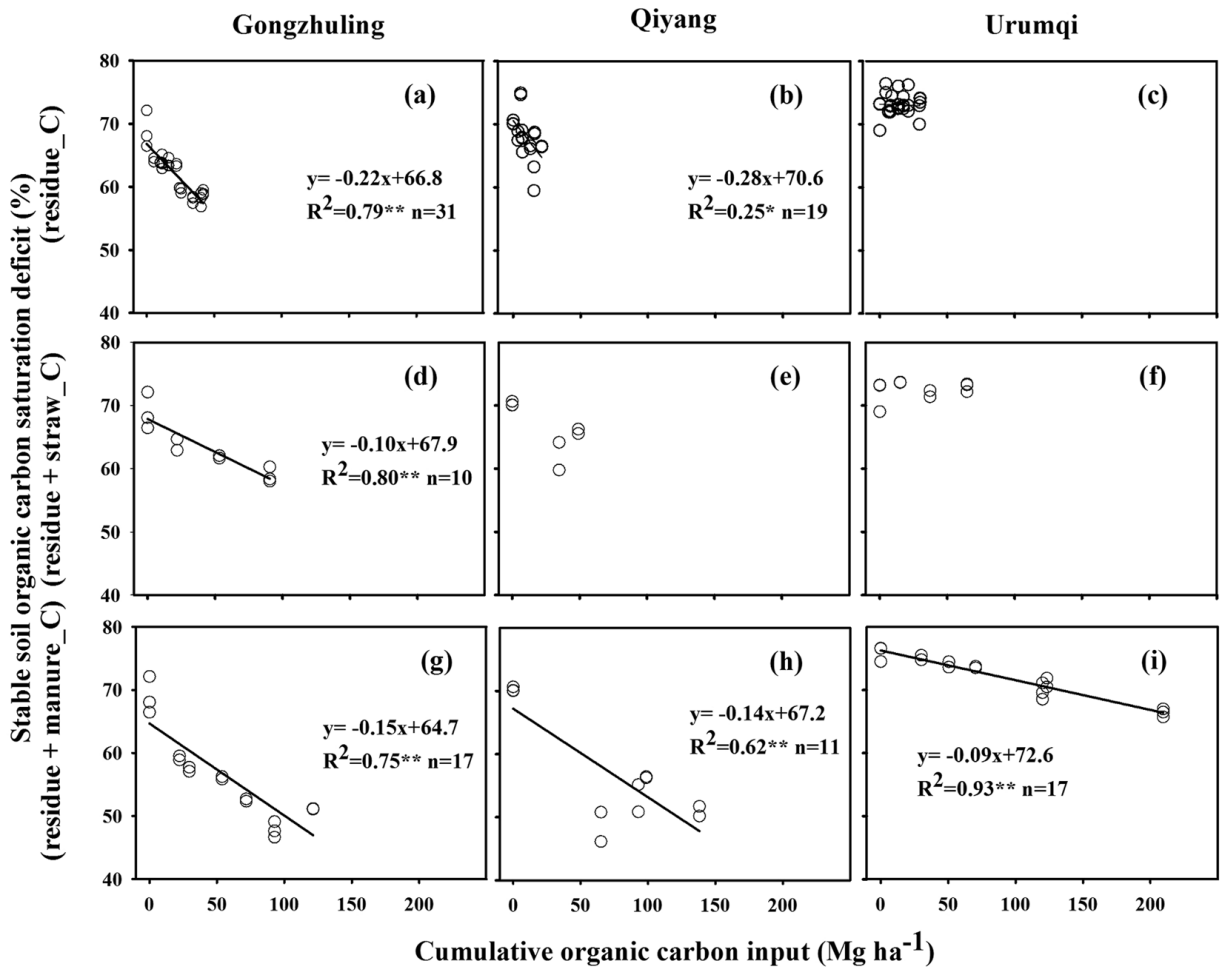
Relationship of the stable C saturation deficit with different ranges and different types of cumulative C input derived from residue-C under CT, N, NP and NPK treatments (**a**–**c**); residue plus straw-C under NPKS (**d**–**f**); and residue plus manure-C under NPKM and hNPKM (**g**–**i**) over 17 years of fertilization at the three long-term sites (*P* < 0.01 denoted by two asterisks and *P* < 0.05 denoted by one asterisks). CT, non-fertilized control; N, mineral nitrogen; NP, mineral N and phosphorus; NPK, mineral N, phosphorus, and potassium combination; NPKS, NPK plus crop straw return; NPKM, NPK plus livestock manure; hNPKM, higher rates of mineral fertilizer and manure input.

The amount of C input is well documented as the dominant factor influencing the total or fractional soil C dynamics, but the effect on fraction soil C dynamics is rarely quantified. Our results showed that the stable soil C_sd_ increased after 17 years under CT at the Qiyang site (Table 2, Fig. 2b), indicating the amount of C input was not sufficient to maintain the initial organic C content of the fine soil fractions and the organic C content of fine soil fractions decreased with fertilization time. In some studies on upland or paddy soils, the minimum carbon input needed to maintain the initial SOC varied according to the soil properties and cropping system^5,30^. Our results suggest that a small amount of C input only maintained the organic C content of fine soil fractions under mineral fertilizer treatments, whereas a large amount of C input, such as that found in manure treatments, is required to stabilize more C to the fine soil fractions. Under a specific climate and management practice, when SOC does not increase over fertilization time, the soil reaches an equilibrium state, but a disturbance or change in management may induce a new increase in SOC until a new C equilibrium state is reached^16^. Although the amount of C input was higher under NPKS than under CT and mineral fertilizer treatments, the stable soil C_sd_ levelled off after a few years of application, indicating that stable SOC under NPKS reached a steady state (Fig. 2d,f). This condition probably arose because soil C inputs and outputs reached equilibrium within the first one to two decades of regular straw return. Other studies also found that straw return was an effective practice to sustain and increase SOC in a short period but could not enhance SOC in the long term^33^. Among the nitrogen fertilizer application, straw return, and no-tillage practices, straw return provided the greatest carbon sequestration in both current and potential scenarios^4^. However, we found that the stable soil C_sd_ dynamics of NPKS were similar to those of CT or mineral fertilizer treatments (Fig. 2a–f) and that the conversion efficiency of straw-C was lower than that of residue-C (Fig. 4a,d). To promote C sequestration of stable SOC, straw-C conversion efficiency needs to be improved. Compared to the soil under straw return treatment, the soil with manure amendments had more C input (Fig. 1). Even after 17 years, the stable soil C_sd_ in the manure treatments declined linearly with fertilization time (Fig. 2g,i), indicating that stable SOC had not yet reached a steady state and soils under the manure treatment can sequester additional C input. Among the variety of fertilizer applications used at our study sites, the manure amendments seem to be the most effective in sequestering C as stable SOC.

Furthermore, this study showed that the stable soil C_sd_ was regulated not only by the quantity but also by the quality of C inputs. At Gongzhuling, the change rate of the stable soil C_sd_ with crop residue-C inputs was larger than that with straw-C or manure-C (Fig. 4a,d,g, Table 2), which indicates that soils with only crop residue-C inputs had a higher conversion rate than those with straw-C or manure-C inputs. The differences probably occurred because these soils received different qualities of C inputs. Sequestering C inputs to fine soil fractions is thought to begin with the transformation of plant-derived C to microbial C, followed by stabilization into the soil mineral matrix^28,34^. Different types of C input vary considerably in their rates of decomposition in soils^7,29^; i.e., the SOC conversion rate is influenced by the litter quality^35^. Through a conceptual assumption^35^, in soils with low C saturation, the litter-C to mineral-associated SOC conversion rate of high-quality litter (high N concentration, low C/N ratio, and low phenol/lignin concentration) exceeded the conversion rate of the low-quality litter. In soils with high C saturation, this pattern was reversed. In our study, for the quality of litter in Gongzhuling site, corn residue or straw-C had a higher C/N than did manure-C, and the average C/N ratios of corn root, corn straw, and pig manure were 82, 89, and 31, respectively^36,37^. However, we found in our study that the soils were far from saturated (with less than 50% saturation) and that the conversion rate of C inputs into fine fractions followed the pattern straw-C < manure-C < residue-C. This pattern was probably related to the larger stable soil C_sd_ in CT and mineral fertilizer treatments than in NPKS or manure treatments.

Another noteworthy finding was the greater significant effect of soil properties on the stable soil C_sd_ at a plot scale. On a field-level scale, soil properties or cropland management practices control the variation of soil C turnover^8,9,30,31^. While the effects of soil properties on stable C sequestration potential have received much attention in recent years^21,26^, there are insufficient quantitative relationships to explain such effects. However, Luo *et al*. (2017) revealed the relative importance of climate, soil properties, C inputs and C pools and their complex interconnections in regulating TOC dynamics^32^. Edaphic factors explained up to 39.7% of the variance in TOC sequestration efficiency (CSE), which was more than that explained by climate factors and C inputs^31^. The authors also identified soil available nitrogen content and pH as the major soil properties explaining CSE variance^31^. We performed quantitative analyses to describe the importance of soil properties and found that soil properties were the main significant factors influencing the stable soil C_sd_ variance (Fig. 3), highlighting the importance of soil properties in stabilizing C in soils. We also found that the soil structure (BD) and soil chemistry (TOC, AP and pH) have significant effects on stable soil C_sd_ (Fig. 3). Furthermore, the C: N: P ratios in crop residue and straw are much larger than those in the soils in our study^36^, suggesting that higher levels of N and P are required for more C stabilization by soil fine fractions under CT, N, NP, NPK, and NPKS treatments. Our results suggested that to stabilize more C inputs, attention should also be paid to the stoichiometry of C inputs and soils.

Among the climate variables, our results suggested that temperature was more important than precipitation in determining the stable soil C_sd_ in the three studied sites. However, the stable soil C_sd_ was positively correlated with MAT and negatively correlated with mean annual precipitation (MAP) at a regional scale in the croplands of southeast Germany^21^. Strong positive correlations were observed between the C sequestration potential and temperature and precipitation in Hebei Province in China^27^. Temperature and precipitation are generally regarded as the dominant factors affecting the soil C turnover time at global or regional scales^38^.The inconsistencies in the effects of temperature and precipitation on stable soil C_sd_ might have arisen because the soil properties of terrestrial ecosystems are controlled by a variety of factors that operate at different scales^39^. Other studies have also shown negative correlations between the effective cumulative temperature and C conversion rate in China^5^. In our study, these agro-ecosystems were mainly temperature limited rather than water limited, resulting in the greater response of stable soil C_sd_ change to temperature (negative effects on the stable soil C_sd_, Table S1) than to precipitation. Overall, stable soil C_sd_ dynamics were controlled by a complex interplay of C inputs and edaphic and climatic conditions. These findings suggested that the impacts of C inputs and edaphic and climatic conditions should be considered together when optimizing fertilization management for stable soil C sequestration. However, the interrelations of stable soil C sequestration with these drivers and their potential connection networks are rarely assessed quantitatively, and they should be evaluated in further studies.

## Conclusions

After 17 years of manure amendment, the stable soil C_sd_ ranged from 47.8% to 59.5% under the NPKM and hNPKM treatments and was higher than that with other treatments at the study sites. This finding suggests that these soils stabilized more C than soils under other treatments but had reached only half C saturation, showing that these soils still can sequester considerable amounts of additional C input. Stable soil C_sd_ dynamics were controlled by a complex interplay of C inputs and edaphic and climatic conditions. Our results show that a small amount of C input only maintained organic C content of soil fine fractions, and that a large amount of C input is required to stabilize more C to soil fine fractions. With different types of C inputs, soils with only residue-C input had higher C conversation rates than soils with straw-C or manure-C input. Additionally, the residue-C amount from the crop is limited, and it is not sufficient to enhance the content of organic C for a long time. The use of organic amendments plus mineral fertilizers is recommended for stable C sequestration and resource utilization management practices. Moreover, our results suggest that to stabilize additional C inputs, it is important to pay more attention to individual soil characteristics and temperature. Overall, these results have important implications for the regulation of the stable C sequestration potential at the field scale.

## Materials and Methods

### Study area and sampling

The characteristics of the three long-term experimental sites are listed in Table 3. These sites are part of the “National Soil Fertility and Fertilizer Effects Long-term Monitoring Network” of China, which was initiated in 1990. The experimental plot sizes of each treatment were 400 m^2^, 468 m^2^, and 196 m^2^ at Gongzhuling, Urumqi, and Qiyang, respectively^5,8^. Each treatment was replicated twice in a randomized block design at Qiyang, whereas there was no field replication of the experimental plots at Gongzhuling or Urumqi. The area of each experimental plot was sufficiently large to represent the local variation of soil properties considering the following soil sampling procedure: during sample collection, five soil samples (0–20 cm depth) were randomly collected from each treatment plot and thoroughly mixed as one pseudoreplicate, and three pseudoreplicates in total were collected from each treatment plot at Gongzhuling and Urumqi. Soil samples were collected after crop harvest and before tillage during September and October 2007. Each soil sample was sieved through a 5-mm mesh screen. Roots and stubble in soil samples were removed by hand. The samples were then air dried for analysis of the soil properties and SOC fractions. Archived soil samples from a 0–20 cm depth that were taken in 1990, 1994, and 2000 at Gongzhuling and Urumqi and in 1990 and 2000 at Qiyang were obtained for analysis of soil properties and SOC fractions (three samples in 1990 as a baseline before the treatments and two samples for each treatment in the other archived years at every site). These crude subsamples were air dried and sealed in glass vials that were free of air to minimize C mineralization and then stored at room temperature as archived soil material until further analysis^40^. All samples were analysed in 2007. The soil type at each site was classified based on the United Nations Food Agriculture Organization (FAO) soil taxonomy system. The MAT and MAP are the mean values for every day. Temperature and precipitation data for each site were collected from the nearest meteorological station of the China Meteorological Administration.

**Table 3:** Geographic location, mean annual precipitation (MAP), mean annual temperature (MAT), soil classification, soil clay type, and cropping systems of the three long-term experimental sites.

Site	Location	MAP (mm)	MAT (°C)	FAO/UNESCO	Clay types	Cropping system
Gongzhuling	43°30′N, 124°48′E	525	4.5	Luvic Phaeozems	2:1	MC (corn)
Qiyang	26°45′N, 111°52′E	1250	18	Ferralic Cambisol	1:1	DC (corn-wheat)
Urumqi	43°58′N, 87°25′E	310	7.7	Haplic Calcisol	2:1	MC (corn/wheat/wheat)

MC: mono-cropping; DC: double-cropping.

### Fertilizer treatments

 At each site, we selected the following fertilizer treatments: (1) non-fertilized control (CT); (2) mineral nitrogen (N); (3) mineral N and phosphorus (NP); (4) mineral N, phosphorus, and potassium combination (NPK); (5) NPK plus crop straw return (NPKS); (6) NPK plus livestock manure (NPKM); and (7) higher rates of mineral fertilizer and manure input (hNPKM). All fertilizers were mixed into the soil to a depth of 20 cm. Details of the mineral fertilizer application rates are shown in Table S2, and the sources and application rates of organic amendments are listed in Table S3.

### Soil sample analysis

Air-dried soil samples sieved through a 2-mm screen were used to determine pH (1:1 w/v water) and for soil particle-size fractionation. Sub-samples were crushed to pass through a 0.25-mm screen for the measurement of total SOC^41^. The total and available nutrient concentrations (N, P) were quantified based on classical analytical methods^42,43^. The size fractionation of soil samples was performed with a slightly modified soil particle-size fractionation method^44,45^. Air-dried soil (10 g) was suspended in 100 mL of water in a 250-mL beaker and then ultrasonicated at 50 KHz for 30 min. The dispersed soil suspension was filtered through a 53-μm sieve until the solution became clear; only the sand fraction (53–2000 μm) was left on the sieve. Further separation of soil particles (<53 μm) to finer soil fractions was performed by centrifugation. Using Stokes’ law, the coarse silt (5–53 μm), fine silt (2–5 μm), coarse clay (0.2–2 μm), fine clay (<0.2 μm), and solution were separated by different centrifugal speeds and centrifugation times as previously described^46^. The soil fractions were dried at 60 °C, weighed, and then crushed to pass through a 0.25-mm screen. The carbon contents of these fractions were determined with the potassium dichromate method^43^. Because our main goal was to compare the dynamics of the soil C saturation deficit (C_sd_) of the fine fractions (all particles <53 μm), we calculated the C content and the proportion of soil mass (mass proportion) of the fine soil fractions (all particles <53 μm) by summing the C content and the mass proportion of the size fractions of coarse silt (5–53 μm), fine silt (5–2 μm), coarse clay (0.2–2 μm), and fine clay (<0.2 μm).

### Calculation of stable soil C saturation deficit

To determine the stable soil C saturation deficit, we calculated the organic C saturation of the fine fractions (<53 μm) using the previously described relationships between the soil texture and C content of fine fractions^19^: C_max_ = 0.21*x* + 14.76 for soils dominated by 2:1 clay (Gongzhuling and Urumqi) and C_max_ = 0.26*x* + 5.5 for soils dominated by 1:1 clay (Qiyang), where *x* is the mass proportion (%) of the fine fractions. The stable soil C saturation deficit (C_sd_, %) was then estimated using the following formula:

1 $${\rm{Stable}}\,{\rm{soil}}\,{{\rm{C}}}_{{\rm{sd}}}=100-{C}_{cur}/{C}_{\max }\times 100$$

where C_cur_ is the current organic C content of the fine fractions.

We calculated the stable soil C_sd_ change rate (%/yr) as:

2 $${\rm{Stable}}\,{\rm{soil}}\,{{\rm{C}}}_{{\rm{sd}}}\,{\rm{change}}\,{\rm{rate}}=\frac{{({\rm{Stable}}{\rm{soil}}{{\rm{C}}}_{{\rm{sd}}})}_{t+{\rm{\Delta }}t}-{({\rm{Stable}}{\rm{soil}}{{\rm{C}}}_{{\rm{sd}}})}_{t}}{{\rm{\Delta }}t}$$

where (stable soil C_sd_)_t_ and (stable soil C_sd_)_t+Δt_ are the stable soil C_sd_ (%) at time t and t + Δt, respectively.

### Estimation of C input

Cumulative C input was estimated as the sum of crop residue-C (*C*_input-crop_) and extra organic amendment C input (manure or straw-C, *C*_input-m/s_) for each treatment from 1990 to the given year.

3 $${C}_{input-crop}=(({Y}_{g}+{Y}_{s})\times R\times {D}_{r}+{R}_{s}\times {Y}_{s})\times (1-{W}_{crop})\times {C}_{crop}$$

4 $${{\rm{C}}}_{\mathrm{input}-m/s}=\frac{{{\rm{C}}}_{m/s}\times {(1-W}_{m/s})\times {A}_{m/s}}{1000}$$

where *Y*_g_ is the biomass of grain (kg ha^−1^), *Y*_s_ is the biomass of straw (kg ha^−1^), *R* is the ratio of belowground biomass to aboveground biomass (26% for corn and 30% for wheat)^47^, *D*_r_ is the ratio of belowground biomass at 0–20 cm to total belowground biomass (85% for corn and 75% for wheat)^48,49^, (*Y*_*g*_ + *Y*_*s*_) × *R* × *D*_*r*_ is the biomass of belowground residue, *R*_s_ is the ratio of residue biomass to straw biomass (3% for corn and 18% for wheat), *R*_*s*_ × *Y*_*s*_ is the biomass of aboveground residue, *W*_crop_ is the water content of grain or straw (14% for corn and 14% for wheat)^30,50^, C_crop_ is the organic C content of crops (444 g kg^−1^ for corn and 399 g kg^−1^ for wheat), *C*_m/s_ is the organic C content of manure or straw (414 g kg^−1^and 382 g kg^−1^for pig manure at Gongzhuling and Qiyang, respectively, 347 g kg^−1^ for goat manure at Urumqi, 444 g kg^−1^ for corn straw, and 399 g kg^−1^for wheat straw), *W*_m/s_ is the water content of manure or straw (69% and 71% for pig manure at Gongzhuling and Qiyang, respectively, 51% for goat manure at Urumqi, 14% for corn straw, and 14% for wheat straw)^36^, and *A*_m/s_ is the application rate of manure or straw (kg ha^−1^). Therefore, organic C input into soil comprised plant residues under the CT, N, NP, and NPK treatments; plant residues plus organic manure under the NPKM and hNPKM treatments; and plant residues plus straw under the NPKS treatment.

### Statistical analysis

Linear regression analysis was performed to determine the relationship between the cumulative C input and stable soil C_sd_. Simple linear regression within groups was used to compare the slopes of the relationships between the cumulative C input and stable soil C_sd_ for different qualities.

Furthermore, we screened the indicators by conducting a lasso regression analysis to identify the quantitative contributions on stable soil C_sd_. Lasso regression analysis is a shrinkage and variable selection method for linear regression models. The analysis involves a type of machine-learning algorithm and biased estimation that can exclude multicollinearity and enable variable selection. Variables with a regression coefficient equal to zero after the shrinkage process are excluded from the model, and variables with non-zero regression coefficients variables are strongly associated with the response variable. Explanatory variables can be either quantitative, categorical or both.

We considered three latent variables, that is, soil properties, climate and C inputs, to assess their effects on stable soil C_sd_ for the Gongzhuling, Qiyang and Urumqi sites. The latent variables were reflected by the 13 predictor variables (Table S4). The data were randomly split into the training set that included 70% of the observations and the testing set that included the remaining 30%. The least angle regression algorithm with folds equal to 3 for cross-validation was used to estimate the lasso regression model in the training set, and the model was validated using the test set to prevent overfitting of the model. The predicted stable soil C_sd_ by the lasso regression model driven by the identified variables was compared with observed stable soil C_sd_ calculated based on Equation (1). This comparison allows us to evaluate the overall predictive power of all considered variables.

Linear regression analyses were performed using SPSS 20 software. Significant differences were determined with the LSD test at the 0.05or 0.01 levels of probability depending on the analysis. Prior to analysis, residuals were checked for homogeneity of variance and normality to ensure that they satisfied the assumptions of parametric tests. The lasso regression analyses were conducted in Python. All graphs were prepared using SigmaPlot 12.0.

## Electronic supplementary material

Supplementary information
