# Supplementary material for: Combinations of soil properties, carbon inputs and climate control the saturation deficit dynamics of stable soil carbon over 17-year fertilizaiton
Source: Sci Rep. 2018 Aug 23;8:12653. doi: 10.1038/s41598-018-31028-x (PMC6107555; doi:10.1038/s41598-018-31028-x)
Supplement: Supplementary file 1 — Supplementary information [file 41598_2018_31028_MOESM1_ESM.pdf]

**Supplementary information for**

# Soil properties, carbon inputs, and climate combine to control the stable soil carbon saturation deficit dynamics over different 17-year fertilizations

Jiaying Di[1, 2], Minggang Xu[1,*], Wenju Zhang[1], Xiaogang Tong[3], Xinhua He[4, 5], Hongjun Gao[6], Hua Liu[7] & Boren Wang[1]

[1] *National Engineering Laboratory for Improving Quality of Arable Land, Institute of Agricultural Resources and Regional Planning, Chinese Academy of Agricultural Sciences, Beijing 100081, China*

[2] *Key Laboratory of Agro-information Services Technology of Ministry of Agriculture, Agriculture Information Institute of Chinese Academy of Agricultural Sciences, Beijing 100081, China*

[3] *College of Resources and Environment, Northwest A & F University, Yangling, Shannxi 712100, China*

[4] *College of Resources and Environment, Southwest University, Chongqing 400715, China*

[5] *School of Plant Biology, University of Western Australia, Crawley, WA 6009, Australia*

[6] *Centre of Agricultural Environment and Resources, Jilin Academy of Agricultural Sciences, Changchun 130033, China*

[7] *Institute of Soil and Fertilizer, Xinjiang Academy of Agricultural Sciences, Urumqi, China*

***Corresponding authors.**

*E-mail address:* xuminggang@caas.cn (M.G. Xu)

**Table S1. Coefficient values of the 13 predictor variable of the lasso regression model at the three long-term experimental sites.** TOC, total soil organic content; TN, total soil nitrogen; AN, available nitrogen; TP, total phosphorus; AP, available phosphorus; BD, bulk density; pH, soil pH; MAP, mean annual precipitation; MAT, mean annual temperature; Cum_T > 10°C, effective cumulative temperature >10°C; Cum_C input, cumulative C input amount C input_R, crop residue-C input amount; C input_M/S, the extra organic C input amount (i.e., manure or straw).

| Category | Predictor variable | Coefficient value | | |
|---|---|---|---|---|
| | | Gongzhuling | Qiyang | Urumqi |
| Soil properties | TOC | -4.63 | -4.19 | -11.82 |
| | TN | -4.18 | 0.00 | 0.00 |
| | AN | 0.00 | 0.00 | 0.00 |
| | TP | 10.13 | 0.00 | 0.00 |
| | AP | -11.30 | 0.00 | 0.00 |
| | BD | 2.23 | 0.00 | 0.00 |
| | pH | 0.00 | 0.00 | 4.02 |
| Climate | MAP | 0.00 | 0.00 | 0.00 |
| | MAT | -1.21 | 0.00 | -3.53 |
| | Cum_T | -0.24 | 2.77 | 0.00 |
| C inputs | Cum_C input | 0.00 | 0.00 | 0.00 |
| | C input_R | -5.28 | 0.00 | 0.00 |
| | C input_M/R | 0.00 | -15.05 | -9.72 |

**Table S2. Application rates of mineral fertilizers at the three long-term experimental sites.** N, mineral nitrogen; NP, mineral N and phosphorus; NPK, mineral N, phosphorus, and potassium combination; NPKS, NPK plus crop straw return; NPKM, NPK plus livestock manure; hNPKM, higher rates of mineral fertilizer and manure input.

| Site | Crops | N/NP/NPK | NPKS | NPKM | hNPKM |
|---|---|---|---|---|---|
| | | Mineral N-P-K (kg $ha^{-1}$ $yr^{-1}$) | | | |
| Gongzhuling | Corn | 165-36-68 | 112-36-68 | 50-36-68 | 75-54-103 |
| Qiyang | Corn | 210-36-70 | 210-36-70 | 63-36-70 | 94-54-105 |
| | Wheat | 90-15-30 | 90-15-30 | 27-15-30 | 40-23-45 |
| Urumqi | Corn/wheat/wheat | 241-59-48 | 217-50-42 | 85-22-10 | 150-39-15 |

**Table S3. Sources and application rates of organic amendments in various fertilizer treatments at the three long-term experimental sites.** NPK, mineral N, phosphorus, and potassium combination; NPKS, NPK plus crop straw return; NPKM, NPK plus livestock manure; hNPKM, higher rates of mineral fertilizer and manure input.

| Site | NPKS | NPKM/hNPKM | NPKS | NPKM | hNPKM |
|---|---|---|---|---|---|
| | Source | | Application rate (Mg $ha^{-1}$ $yr^{-1}$) | | |
| Gongzhuling | CS | PM | 7.5 | 23 | 34.6 |
| Qiyang | WS | PM | Half[&] | 29 | 44 |
| | CS | PM | Half[&] | 13 | 19 |
| Urumqi | CS/WS | GM | All[&] | 30 | 60 |

CS: corn straw, WS: wheat straw, PM: pig manure, GM: goat manure. [&]: Half or all crop straw of the previous season was returned back to the soil.

**Table S4. The 13 predictor variables of the lasso regression model at the three long-term experimental sites.**

| Category | Predictor variable | Description | Unit |
|---|---|---|---|
| Soil properties | TOC | total soil organic carbon | g $kg^{-1}$ |
| | TN | total soil nitrogen | g $kg^{-1}$ |
| | AN | available nitrogen | g $kg^{-1}$ |
| | TP | total phosphorus | g $kg^{-1}$ |
| | AP | available phosphorus | mg $kg^{-1}$ |
| | BD | bulk density | g $cm^{-3}$ |
| | pH | soil pH | |
| Climate | MAP | mean annual precipitation | mm |
| | MAT | mean annual temperature | ℃ |
| | Cum_T | effective cumulative temperature >10℃ | ℃ |
| C inputs | Cum_C input | cumulative C input amount | Mg $ha^{-1}$ |
| | C input_R | crop residue C input amount | Mg $ha^{-1}$ |
| | C input_M/R | extra organic C input amount include manure or straw | Mg $ha^{-1}$ |

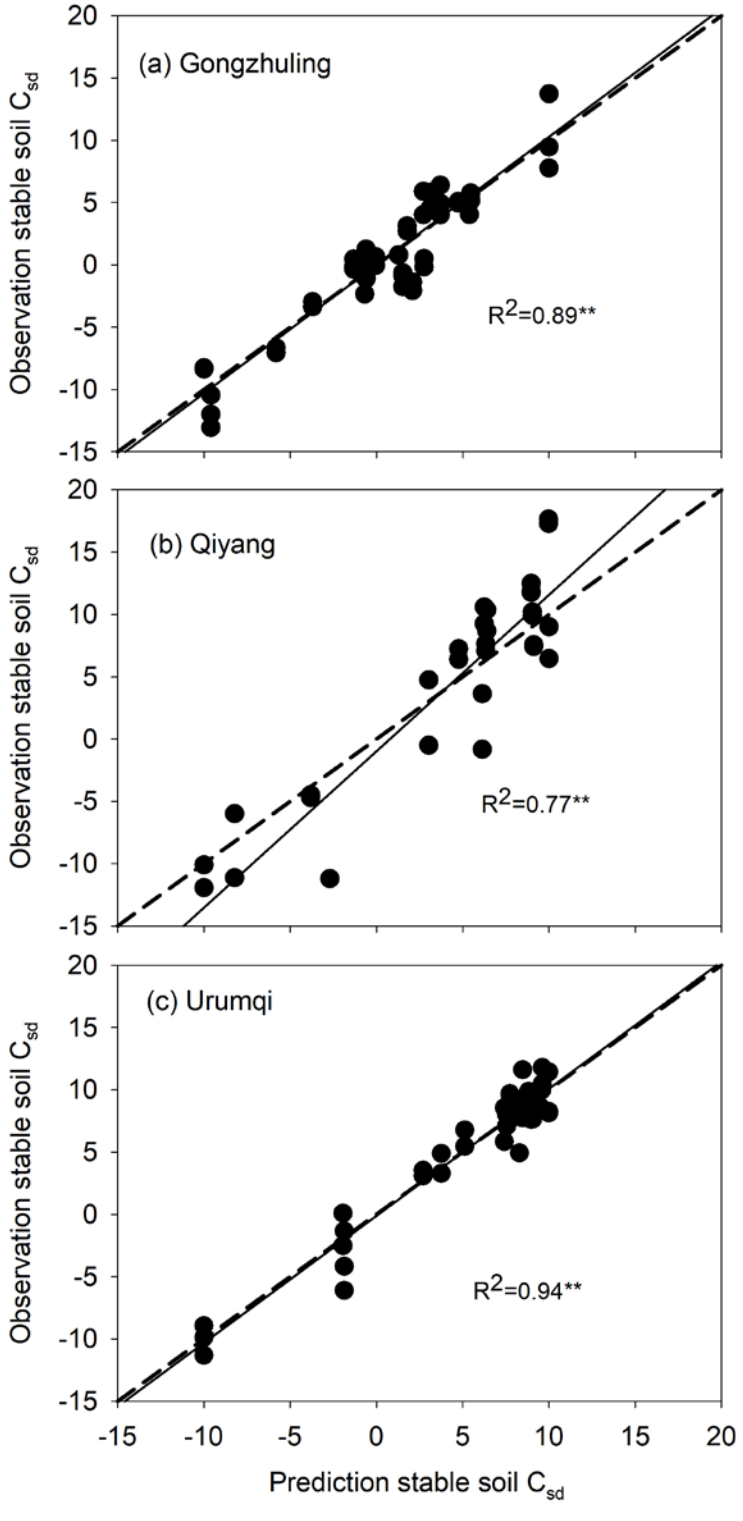


Figure S1. Observed values and values predicted by the lasso linear regression model using the predictors shown in Fig. 3. The dashed line shows the 1:1 line (in all sites *P* < 0.01, denoted by two asterisks).
